# History of intestinal transplantation in Australia

**DOI:** 10.1016/j.intf.2025.100072

**Published:** 2025-07-11

**Authors:** Katrina Tan, Brooke Chapman, Darren Wong, Jason Yap, Graham Starkey, Adam Testro

**Affiliations:** a The Australian Intestinal Transplant Service, Melbourne, Australia; b The Royal Children’s Hospital, Parkville, Australia; c Department of Surgery (Austin Precinct), The University of Melbourne, Australia; d Australian Centre for Transplantation Excellence and Research, Melbourne, Australia

**Keywords:** Intestinal failure, Intestinal Transplantation, Parenteral Nutrition, Nutrition

## Abstract

Intestinal transplantation was introduced into Australia in 2010 to meet the need of a small number of patients with irreversible intestinal failure who would otherwise need to travel abroad to access this life-saving therapy. Due to small case numbers, a single center based in Melbourne, Victoria, services all of Australia and New Zealand. Centralization of clinical activity has resulted in excellent patient outcomes, with 5-year graft and patient survival matching that of the more experienced international centers. However, on the path to establishing a successful service, multiple significant challenges were encountered, including training of staff, surgical and immunosuppression protocol development, and donor organ acceptance and recipient allocation based on the vast geography of Australia. Deficiencies in intestinal failure management on a national level have posed some further concerns regarding timely access to and referral for transplantation. Herein we discuss the establishment of an intestinal transplant service in Australia, how challenges were overcome and what barriers remain.

## Introduction

Intestinal transplantation was introduced into Australia in 2010 to meet the need of a small number of patients with irreversible intestinal failure who would otherwise need to travel abroad to access this life-saving therapy. Introducing a new, highly specialized service into a geographically vast country posed significant challenges. Herein we report the history of intestinal transplantation in Australia; the past, the present and the future.

## Demographics and healthcare systems in Australia

Located in the Southern Hemisphere, between the Indian and Pacific Oceans, Australia is the 6th largest country in the world by total area (7692,024 km^2^), with a land mass approximately 80 % of that of the United States of America. The current population of Australia is 27.3 million people, which is projected to grow to 31.3 million over the next decade[1]. Whilst Australia is geographically vast, 85 % of the population live in urban areas, with approximately 50 % of the population along the eastern and southeastern coastline.(1) Australia consists of six states (New South Wales, Victoria, Queensland, Western Australia, South Australia and Tasmania), and two major mainland territories (Australian Capital Territory and the Northern Territory).

Healthcare in Australia is a mixture of private and public medicine. *Medicare* is Australia’s public health insurance system, providing largely outpatient healthcare to eligible Australian residents at low or no cost. The *Pharmaceutical Benefits Scheme* (PBS) provides subsidized prescription medicines making pharmaceuticals more affordable and accessible. Whist Medicare and the PBS are funded by the federal government, the public hospital system is funded by individual states and territories. Transplantation governance also sits with the federal government, however the actual provision of transplantation services within Australia remains the responsibility of the individual state governments. Not all states and territories of Australia provide all solid organ transplants. Some states outsource the provision of transplant services for their residents to another state that may have a more well-developed service, and cross-border financial cost recovery arrangements exist to accommodate this.

## Structure of Australian transplant services

Abdominal transplant services in Australia are state based except for Tasmania, which currently doesn’t provide any transplantation and has a longstanding referral relationship with Victoria. Likewise, the Northern Territory refers patients to South Australia and the Australian Capital Territory to New South Wales. Whilst liver transplantation is accessible in five states*, The Australian Intestinal Transplant Service* based in Victoria, is the only active intestinal transplant service in Australia, providing intestinal transplantation to all of Australia and to New Zealand.

The Australian Organ and Tissue Donation and Transplantation Authority (OTA) was established in 2009. OTA delivers a nationally coordinated program to increase organ and tissue donation to improve opportunities for transplantation in Australia. The national *DonateLife* program is delivered in partnership with the *DonateLife Network* and state and territory governments. Unlike other transplant jurisdictions abroad, organs are in general allocated within the state from which they arise. The exception to this is for urgent listings, such as acute liver failure, where there are agreements in place for cross-border sharing of organs according to strict criteria defined by the Transplantation Society of Australia and New Zealand (TSANZ) [2].

The governance surrounding intestinal transplantation sits with the TSANZ Liver and Intestinal Transplant Advisory Commitee (LITAC). LITAC is the peak clinical advisory body for issues related to liver and intestine donation and transplantation in Australia and consists of representatives from all liver transplant units and the intestinal transplant unit. LITAC reports through the TSANZ Council to OTA.

As there is a single intestinal transplant service for the entire country, suitable intestinal grafts are often referred from interstate to the national center in Victoria. The vast land mass of Australia however limits what organ offers can be accepted based on travel time and hence predicted cold ischemia times. In practice therefore, most intestinal grafts arise from the eastern seaboard (Queensland, New South Wales, Victoria) as well as the island state of Tasmania.

## Intestinal failure

Intestinal failure (IF) management in Australia differs significantly between adult and pediatric services. Pediatric intestinal failure care is centralized to a small number of specialized IF centers with multidisciplinary teams, but unfortunately adult IF management suffers from a lack of centralization of care, which in turn leads to significant variation in practice and expertise, a less clear referral pathway for intestinal transplantation, and potentially poorer patient outcomes.

Home parenteral nutrition (HPN) has been available in Australia since the late 1970’s. Whilst most large centers have a multidisciplinary team involved in patient care this is not universal, and many centers lack adequate resourcing [3], [4]. Further, until recently there has been no functional HPN registry in Australia and no regular benchmarking between units. The Australian HPN Registry, launched in 2024, will provide accurate recording of HPN usage and resource allocation and allow benchmarking within and beyond Australia. Our experience has been that many patients requiring intestinal transplantation are referred late in their disease course, with impending complete loss of venous access, significant liver disease, or severe fistulizing intra-abdominal disease and sepsis. Unfortunately, we have little doubt that transplant-eligible patients may not even get referred for transplantation due to a general lack of awareness of an intestinal transplant service in Australia.

Australia gained access to the glucagon-like peptide 2 (GLP-2) analogue teduglutide for use in adult patients in 2018, initially as part of a product familiarization program, but later in October 2019 as part of the Australian PBS. Teduglutide became available for pediatric patients in Australia in 2023. Teduglutide has made a significant impact on patients with short bowel syndrome-intestinal failure, and we have managed a small number of patients who have derived significant benefit from this novel drug therapy in reducing or eliminating their reliance on parenteral support [5], [6], [7]. It remains to be seen what impact GLP-2 analogues have on the need for transplantation in the medium-to-long term.

## Intestinal transplantation – clinical activity

The Australian Intestinal Transplant Service is a collaboration between Austin Health and the Royal Children’s Hospital Melbourne, established parallel to a longstanding and highly successful combined liver transplant program. The liver transplant program has been active for over 30 years, performing 90–100 liver transplants annually. Whilst adult patients receive transplantation at Austin Health, and pediatric patients at the Royal Children’s Hospital, there is a sharing of staff, clinical protocols and research endeavours between both institutions. Further, local pediatric transplant recipients are ultimately transitioned to adult care at Austin Health upon turning 18 years of age.

Intestinal transplantation was introduced to Australia in 2010 when the first adult recipient underwent a liver-intestinal transplant at Austin Health [8]. Prior to 2010, a small number of Australians received intestinal transplantation abroad at enormous cost to the Australian Federal Government under the Medical Treatment Overseas Program (MTOP) [9]. The MTOP covers the cost of the medical treatment, airfares and accommodation for approved Australians who need life-saving medical treatment that is only available overseas. Following the initial successful case in Australia in 2010, access to the MTOP for intestinal transplantation closed, and subsequently all Australians requiring this treatment were referred to The Australian Intestinal Transplant Service in Melbourne.

The first pediatric intestinal transplant (liver-intestine) occurred in 2012, the first multivisceral transplant in 2016, and the first modified multivisceral transplant in 2021. Consistent with developing a new service within a country with an existing burden of intestinal failure associated complications, many of the initial referrals to our service had advanced liver disease, hence the bias in our series towards liver-containing grafts.

A retrospective review of the Australian Intestinal Transplant Service database was performed capturing all patients assessed for intestinal transplantation between January 1, 2009, and May 10, 2025. The study was approved by the Austin Health Human Research Ethics Committee (VicTRI-19485). Thirty-six patients have been assessed for intestinal transplantation. Currently, 17 intestinal transplants have been performed in 16 recipients, with 9 of these occurring in the past 5 years consistent with our cumulative experience and the more widespread awareness of the service driving an increased referral rate. There have been 2 isolated intestinal transplants, 8 liver-intestine transplants, 4 multivisceral transplants, and 3 modified multivisceral transplants. Colon is routinely transplanted if possible. Indications for transplantation were short-bowel syndrome n = 7 (41.2 %), dysmotility n = 3 (17.6 %), portomesenteric venous thrombosis n = 3 (17.6 %), desmoid tumour n = 3 (17.6 %), and a single (5.9 %) re-transplant following graft loss from severe rejection.

**Fig. 1:**
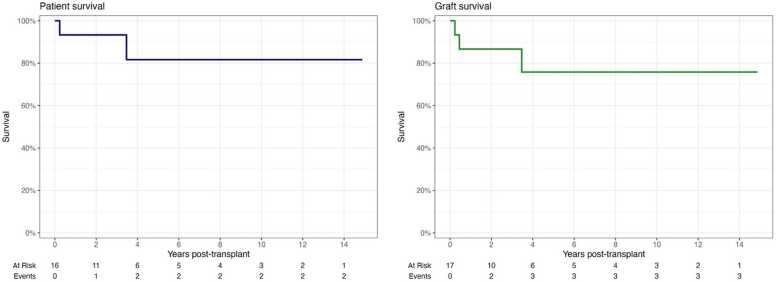
Patient and graft survival post intestinal transplantation. Data collected prospectively as part of routine service audit and is complete for all patients. Graphs constructed using the Kaplan-Meier methodology using R v4.5.0.

Graft and patient survival rates are comparable with more experienced international centers (Fig. 1). One graft (5.9 %) has been lost to severe acute T-cell mediated rejection. There has been one case of post-transplant lymphoproliferative disease and one case of graft-versus-host disease, both of which were successfully managed. 93 % of surviving patients have full enteral autonomy. Six patients remain active on the waiting list for transplantation, four patients died whilst on the waiting list and the remaining 10 patients were not put forward for transplantation following assessment.

Throughout the history of intestinal transplantation in Australia, we have used a common immunosuppression regimen consisting of basiliximab induction (with a second dose on post-operative day 4), intravenous methylprednisolone given at induction and immediately prior to graft reperfusion, with subsequent slow weaning over the next 3 months, mycophenolate mofetil and tacrolimus. In line with international practice there is routine anti-microbial and anti-fungal prophylaxis and universal CMV prophylaxis (ganciclovir initially followed by valganciclovir once enteral autonomy is established). Surveillance endoscopy at our service is performed by a small number of gastroenterologists with extensive experience with intestinal allografts, on a routine basis for the first 6 months (twice weekly in month 1, weekly in month 2, fortnightly in month 3, and monthly through until month 6). Beyond month 6 biopsies are performed only on clinical need. Histopathological examination is performed by a small pool of gastrointestinal histopathologists who have undertaken further training abroad in interpreting intestinal allograft histology. Stomas are closed between 6 and 12 months post-transplant where possible.

It is an expectation of the service that interstate patients reside locally for at least the first three months post-transplant, following which they are transferred back to their state-based liver transplant center for ongoing care in a shared care arrangement with our unit. Routine clinical visits are conducted by the local unit, with an annual visit to our unit, either in person or by telehealth. In the event of a significant transplant-related event, the patient is transferred back to our center for ongoing management.

## Gaining transplant expertise

One of the predicted barriers to establishing a new highly specialized service was gaining appropriate levels of training, expertise, and governance. One of the more notable pillars of success in developing intestinal transplantation in Australia was the strong collaboration forged with higher throughput, more experienced international centers. Facilitated through the International Intestinal Failure and Transplant Association (IIRTA), many of our team members have undertaken further training and gained valuable clinical expertise abroad.

The founding surgeons and transplant physicians were trained at the University of Pittsburgh Medical Center (UPMC), Pittsburgh, PA, USA, and Birmingham Children's Hospital, Birmingham, UK. As such our initial clinical protocols have been an evolving amalgam of both of these high performing units. Other members of the core medical staff trained at The Cambridge Centre for Intestinal Rehabilitation and Transplant, Cambridge, UK, and The Transplant & Regenerative Medicine Centre at Sick Kids, Toronto, Canada. Aside from these clinicians, our expert clinical dietician, our histopathologists and senior nursing staff have each undertaken brief periods of training at UPMC, Pittsburgh and Mount Sinai Medical Centre, New York.

Two individuals played a significant role in establishing intestinal transplantation in Australia and are deserving of specific recognition. Both Assistant Professor Geoffrey Bond from UMPC, Pittsburgh and Professor Darius Mirza from Birmingham, UK undertook clinical sabbaticals at our institution and assisted with protocol development. Professor Mirza was instrumental as he assisted in performing the first intestinal transplant during his visit in 2010.

As our local caseload grows and our cumulative experience expands, our fellows, both surgical and medical, now obtain excellent clinical exposure to this small cohort of patients and are acquiring a strong knowledge base around the optimal way to care for these highly complex patients.

## Challenges

Australia is actively developing new models of care for IF, particularly in the adult field, and under the direction of the Australasian Society of Parenteral and Enteral Nutrition is developing an HPN registry. Whilst this will no doubt improve IF care in the longer term, it is also likely to address one of the biggest ongoing challenges we face, which is raising awareness of the service in Australia, and better defining referral pathways so that more Australians can access this lifesaving treatment.

With regards to surgical challenges, abdominal wall transplantation has yet to be performed in Australia and remains the final surgical frontier. There are, however, a number of patients active on our intestinal transplant waiting list with complex Crohn’s disease who will require abdominal wall transplantation as part of their graft.

One of our greatest challenges is the vast land mass of Australia which is currently served by a single intestinal transplant service, with the implication that both recipients and donors are often remote from our center. Due to low case numbers, the establishment of a second service is unlikely. Under the current circumstances, the process of assessing and working up a patient for transplantation is complex and time consuming. Patients need to attend our center for a number of weeks to complete the medical work-up and listing process but then return to their homes whilst awaiting transplantation. At the time of transplant, coordinating travel arrangements for the recipient, based on commercial flight availability, can be problematic and needs significant coordination with regards to timing of donor procurement, which is similarly challenging given concerns with cold ischemia times. Donor offers are not infrequently declined based solely on the logistics of travel time. While not currently clinically available, expansion of novel machine perfusion techniques into the intestinal transplant field may in the future mitigate some of these challenges.

Whilst one of the initial challenges of providing expertise in the surgical and medical management of intestinal transplantation has been overcome, we are now in the fortunate position of developing our own, locally trained fellows who can lead intestinal transplantation in Australia into its next chapter.

## CRediT authorship contribution statement

**Katrina Tan:** Writing – review & editing, Writing – original draft, Data curation. **Brooke Chapman:** Writing – review & editing, Data curation. **Darren Wong:** Writing – review & editing, Formal analysis, Data curation. **Jason Yap:** Writing – review & editing, Data curation. **Graham Starkey:** Writing – review & editing, Data curation. **TESTRO ADAM:** Writing – review & editing, Writing – original draft, Validation, Supervision, Resources, Methodology, Data curation.

## Patient’s/guardian’s consent

Not applicable

## Ethical clearance

Not required

## Funding

No funding was received

## Declaration of Competing Interest

The authors declare that they have no known competing financial interests or personal relationships that could have appeared to influence the work reported in this paper.
